# Probiotics and Human Milk Differentially Influence the Gut Microbiome and NEC Incidence in Preterm Pigs

**DOI:** 10.3390/nu15112585

**Published:** 2023-05-31

**Authors:** Valeria Melendez Hebib, Diana H. Taft, Barbara Stoll, Jinxin Liu, Lee Call, Gregory Guthrie, Nick Jensen, Amy B. Hair, David A. Mills, Douglas G. Burrin

**Affiliations:** 1 USDA Children’s Nutrition Research Center, Department of Pediatrics, Baylor College of Medicine, Houston, TX 77030, USA; valeria.melendez@bcm.edu (V.M.H.); bstoll@bcm.edu (B.S.); lee.call@alumni.bcm.edu (L.C.); gguthrie@bcm.edu (G.G.); 2 Foods for Health Institute, University of California, Davis, CA 95616, USA; dianataft@ufl.edu (D.H.T.); jxnliu@njau.edu.cn (J.L.); yenjensen@ucdavis.edu (N.J.); damills@ucdavis.edu (D.A.M.); 3 Department of Food Science and Technology, University of California, Davis, CA 95616, USA; 4 Laboratory of Gastrointestinal Microbiology, Jiangsu Key Laboratory of Gastrointestinal Nutrition and Animal Health Science and Technology, Nanjing Agricultural University, Nanjing 210095, China; 5 Section of Neonatology, Departments of Pediatrics, Baylor College of Medicine, Texas Children’s Hospital, Houston, TX 77030, USA; abhair@bcm.edu

**Keywords:** microbiome, necrotizing enterocolitis, premature infant, human milk, *Clostridium sensu stricto 1*, *Clostridium perfringens*, human milk oligosaccharide, *Bifidobacterium longum* subsp. *infantis*

## Abstract

Necrotizing enterocolitis (NEC) is the leading cause of death caused by gastrointestinal disease in preterm infants. Major risk factors include prematurity, formula feeding, and gut microbial colonization. Microbes have been linked to NEC, yet there is no evidence of causal species, and select probiotics have been shown to reduce NEC incidence in infants. In this study, we evaluated the effect of the probiotic *Bifidobacterium longum* subsp. *infantis* (*BL. infantis*), alone and in combination with a human milk oligosaccharide (HMO)—sialylactose (3′SL)—on the microbiome, and the incidence of NEC in preterm piglets fed an infant formula diet. We studied 50 preterm piglets randomized between 5 treatments: (1) Preterm infant formula, (2) Donor human milk (DHM), (3) Infant formula + 3′SL, (4) Infant formula + *BL. infantis*, and (5) Infant formula and *BL. infantis* + 3′SL. NEC incidence and severity were assessed through the evaluation of tissue from all the segments of the GI tract. The gut microbiota composition was assessed both daily and terminally through 16S and whole-genome sequencing (WGS) of rectal stool samples and intestinal contents. Dietary *BL. infantis* and 3′SL supplementation had no effect, yet DHM significantly reduced the incidence of NEC. The abundance of *BL. infantis* in the gut contents negatively correlated with disease severity. *Clostridium sensu stricto 1* and *Clostridium perfringens* were significantly more abundant in NEC and positively correlated with disease severity. Our results suggest that pre- and probiotics are not sufficient for protection from NEC in an exclusively formula-based diet. The results highlight the differences in microbial species positively associated with both diet and NEC incidence.

## 1. Introduction

Necrotizing enterocolitis (NEC) is the leading cause of death resulting from gastrointestinal disease in premature infants, with a mortality rate of 15–40% [1] and an incidence of up to 12% among neonates born with a very low birth weight (<1.5 kg). The etiology of NEC is not completely understood, which limits current approaches to prevent and treat this disease. The most widely known preventive approach against NEC is feeding human breast milk. In the absence of their mother’s own milk, infants receive pasteurized donor human milk (DHM) that is usually obtained from human milk banks. Although human breast milk has been shown to reduce the risk of NEC significantly [2,3], the mechanisms through which it does so remain partially unknown. Currently, treatment for NEC includes broad-spectrum antibiotics, bowel rest, parenteral nutrition, and surgical resection of necrotic sections of the bowel. A more detailed understanding of the pathogenesis of NEC is the key to developing more specific prophylactic and treatment approaches for this disease. The three major risk factors associated with NEC are prematurity, formula feeding, and microbial colonization of the gastrointestinal (GI) tract [4]. Experimental evidence from human and animal studies suggests that the severe intestinal inflammation observed in NEC reflects an immature immune system reacting to the bacterial colonization of an underdeveloped gut [5,6,7]. While is insufficient evidence to implicate a sole species in the pathogenesis of NEC, the identification of distinct fecal microbiota signatures in healthy and diseased infants suggests the gut microbiota might be a target to prevent the morbidity and mortality associated with NEC [8,9,10]. Recent research has highlighted the potential of human milk oligosaccharides (HMOs) [11,12] and probiotics [13] to alter the gut microbiome composition and reduce the risk of NEC. Studies evaluating the effectiveness of HMOs in preventing NEC in animal models have yielded promising results in rat pups while being unsuccessful in preterm piglets, suggesting that further investigation is needed to ascertain the benefits of HMOs in the context of NEC [14,15,16,17]. The administration of certain probiotic formulations has resulted in a reduction in NEC cases in infant cohorts. Among the probiotics tested for the prevention of NEC, *Lactobacillus* spp. and *Bifidobacterium* spp. have been associated with a significantly reduced incidence of severe NEC [18,19,20,21,22]. Similar to the use of HMOs, further investigation concerning the effect of probiotics on the infant microbiome and risk for NEC is needed due to the published infant cohort studies having confounding factors that limit the generalizability of the results, including wide ranges in probiotic dosage, gestational ages, enteral feeding regimes, and the particularities of clinical care.

There is a need to comprehensively evaluate the effectiveness of HMOs and probiotics and their effect of gut microbiome composition in a controlled experimental setting, while reducing confounding factors that may affect the outcomes of the study. The probiotic *Bifidobacterium longum* subsp. *infantis* (*BL. infantis*) has been regarded as effective in the prevention of NEC incidence in several studies [22]. Given that HMOs are well known substrates that enhance the growth and health benefits of *Bifidobacterium* species, in the present study we evaluated the effects of the dietary supplementation of human infants using formula with *BL. infantis* and the HMO 3-sialyllactose (3′SL), both alone and in combination, on the taxonomic composition of the intestinal microbiome and the incidence of NEC in a controlled setting using preterm piglets. We also included a group of piglets fed donor human milk to serve as a positive control, given the well-established evidence that preterm infants fed human milk are protected against the development of NEC. 

## 2. Materials and Methods

### 2.1. Delivery, Clinical Care, and Nutrition

**Figure 1:**
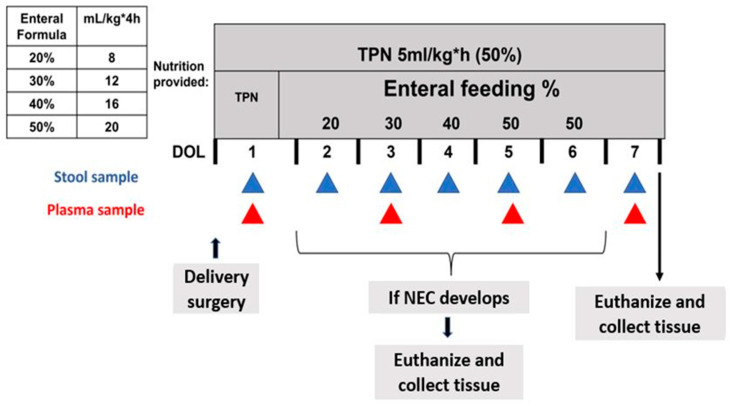
Piglet feeding and sampling regime.

The animal protocol was approved by the Institutional Animal Care and Use Committee (IACUC) of Baylor College of Medicine and was conducted in accordance with the National Institutes of Health (NIH) guidelines. Five pregnant mixed-breed sows were brought to our facility and allowed to acclimate one week before surgery. Preterm piglets were delivered through cesarean section at 104 days of gestation (full term = 114 days), as described previously [23]. A few hours after birth, the piglets were surgically implanted with a jugular venous catheter (0.03″ ID, silastic) and an orogastric tube (6 French Tygon) to facilitate parenteral nutrition and enteral feeding. The piglets received 50% of their total nutritional requirements through total parenteral nutrition (TPN) (120 mL/kg*d, 410 kJ energy/(kg*d), 12.5 g dextrose/(kg*d), 6.5 g AA/(kg*d), 2.5 g fat/(kg*d) (Intralipid) (Sigma-Aldrich St. Louis, MO, USA) at a rate of 5 mL/kg*h via the jugular venous catheter from day 1 until the end of the study on day 7. Beginning on day 2, every 4 h the piglets were fed enterally through their orogastric tube. Enteral feeding began at 20% of the total nutritional requirement (8 mL/kg*4 h) and progressed gradually to 30, 40, and 50% (12, 16, and 20 mL/kg*4 h) (See Figure 1). Passive immunity was provided to the piglets 6, 12, and 24 h after birth through the administration of sow plasma (at a rate of 4, 5, 7 mL/kg) collected at the time of the cesarean section. 

A total of 50 piglets were randomly assigned into five feeding groups. Piglets in the formula (Form) (n = 10) group were fed Enfamil Premature High Protein Liquid Formula (Mead Johnson, Chicago, IL, USA). Piglets in the *Bifidobacterium infantis* (FormBI) (n = 11) group were fed formula (as described above) and 10^9^ colony forming units (CFU)/day. To obtain this mixture, 1 × 10^9^ CFU of *BL. infantis* (0.16 g) was mixed with 3 mL of formula every day. Piglets in the Form3SL (n = 10) group were fed infant formula supplemented with a daily dose of 1.2 g/kg of the human milk oligosaccharide (HMO) sialyllactose (3′SL) while piglets in the FormBoth (n = 10) group were fed the same diet as Form3SL piglets with the addition of a daily oral dose of 1 × 10^9^ CFU of *BL. infantis.* Finally, piglets in the donor human milk (DHM) (n = 9) group were fed pasteurized human milk (Prolacta Bioscience, Duarte, CA, USA). To equalize the carbohydrate content of the formula-fed groups, the diet of both the Form and FormBI groups was supplemented with 1.2 g/kg carbohydrate consisting of 60% corn syrup solids (Grain Processing Corporation, Muscatine, IA, USA) and 40% lactose (Sigma-Aldrich, St. Louis, MO, USA). The piglets that received the probiotic treatment (*BL. infantis*) were housed in separate rooms from the pigs that did not receive probiotic treatment. In order to prevent probiotic contamination, the piglets that received no probiotic treatment were always fed before the piglets that were given probiotic treatment following an appropriate change of personal protective equipment. 

### 2.2. NEC Monitoring

During the 7-day study, piglets were monitored for clinical symptoms of necrotizing enterocolitis (NEC), namely abdominal distension, lethargy, cyanosis, or bloody diarrhea. When piglets developed signs of NEC, the SpO2 was monitored every 3 h, and if the readings dropped below 80% for more than 3–4 h, the piglets were euthanized. Otherwise, the piglets were euthanized at the end of the study on day 7.

### 2.3. Sample Collection

Upon euthanasia, tissue sections of the stomach, proximal jejunum, distal ileum, and colon were collected and frozen or fixed using both formalin and OCT for further analysis. Plasma samples were collected from each piglet at the time of euthanasia. All tissue was assessed at necropsy macroscopically for the generation of a gross NEC score of 1 to 6. The NEC score is determined through the assessment of damage to the major gastrointestinal (GI) regions (stomach, jejunum, ileum, and colon) based on the pathological signs of NEC, as previously described [24]. A score of 1–2 indicates normal healthy tissue, 3–4 indicates moderate redness and inflammation, and 5–6 indicates visible pneumatosis and necrosis (Supplementary Figure S3). A total NEC score is generated by adding the severity scores from the stomach, jejunum, ileum, and colon (4–24). 

Tissues from all the sections of the same major GI regions were fixed and stained using hematoxylin and eosin (H&E) stain for the generation of a histological NEC score and analysis of villus height and crypt depth, as previously described [24]. A histological NEC score from 1 to 5 was assessed through a microscopic evaluation of the tissue damage. In the small intestine (jejunum and ileum sections), a score of 1 indicates no damage, a score of 2 indicates epithelial cell lifting/separation, a score of 3 indicates necrosis of epithelial cells and blunting villi, a score of 4 indicates villi necrosis and some pneumatosis, and a score of 5 indicates transmural necrosis, scant villi, and widespread pneumatosis. In the colon sections, a score of 1 indicates healthy tissue, a score of 2 indicates minimal mucosal breakage, a score of 3 indicates mucosal sloughing with cellular infiltration, a score of 4 indicates pneumatosis with mild mucosal necrosis, and a score of 5 indicates pneumatosis with transmural necrosis. Histological NEC scores were determined by a single observer who was blinded to the treatment groups. Morphometry metrics, including villus height and crypt depth, were measured using ZEISS ZEN Microscopy Software 3.3.

For the analysis of the stool microbiome, rectal, fecal swabs were collected daily and frozen at −80 °C for microbial profile sequencing of the 16S rRNA gene and qPCR assay of *BL. infantis*. When the pigs were euthanized, samples of the small intestine and colon contents were collected for the assessment of the microbial composition through 16S rRNA gene sequencing. The colon content samples were used for whole-genome sequencing (WGS). 

### 2.4. Microbiome Analysis

Approximately 200 mg of stomach, small intestinal, and colon contents, as well as the rectal swabs, were transferred to bead bashing tubes with beads and 750 µL of ZYMOBiomics Lysis Solution (Zymo Research, Irvine, CA, USA). The samples were processed in sets of 12 using a Disruptor Genie TM FastPrep24 set to 6.5 m/s. Each sample was beaten for 5–60 s intervals with five minutes on ice between intervals. The samples were pelleted at 10,000× *g* for 1 min and stored at 4C until a set of 96 samples was prepared. Later, 200 µL of the supernatant was transferred to a 96-well plate, with one sample per well. DNA was extracted using the KingFisher Flex (Thermo Fisher Scientific, Waltham, MA, USA) and the ZYMOBiomics 96 MagBead DNA kit (Zymo Research). A total of 75 µL of MagBeads was used per sample; otherwise, DNA extraction was performed according to the manufacturer’s instructions. 

For 16S rRNA gene amplicon sequencing, the V4 region was amplified as described in Huda et al. [25]. Bioinformatic processing was completed using DADA2 [26] as implemented in QIIME2-2018.11 [27]. Whole metagenome sequencing and trimming were performed on the colon content samples as previously described by Liu et al. [28]. Species’ identification was completed using metaphlan2 [29]. The raw sequence data files have been submitted to the NCBI BioProject database under the accession number PRJNA948045.

### 2.5. Bifidobacterium Longum Quantification in Colon Content Samples

The DNA extracted from the colon content samples as described above was used to quantify the amount of *B. longum* present in the colon using quantitative real-time PCR using PowerUp SYBR Green Master Mix (Applied Biosystems, Waltham, MA, USA). To calculate the colony forming units (CFU) per milligram (mg) of contents, a culture of *B. longum* was diluted 1:10 until a final concentration of 10^5^ CFU/mL was achieved. A standard curve was generated using the DNA from the standard samples. See Table 1 for the primer sequences used.

### 2.6. Evaluation of Pro-Inflammatory Cytokine Expression in Distal Ileum Tissue

A quantitative real-time PCR was performed on the samples of distal ileum tissue. Total RNA was extracted from the tissue using TRIzol (Invitrogen, Waltham, MA, USA) and a commercial RNA extraction kit (Qiagen, Hilden, Germany). Total RNA at a concentration of 350 ng/µL was used to perform reverse transcription to obtain cDNA using a commercially available kit (Applied Biosystems). Both reverse transcription and qRT-PCR were performed on a BioRad CFX96 instrument. qPT-PCR was performed using PowerUP SYBR Green Master Mix (Apploed Biosystems) and primers specific for swine IL-1^β^, TNF-α, IL-8, and IL-17A. The relative mRNA expression was determined relative to the constitutively expressed ^β^-actin using the 2^ΔΔCT^ method. 

### 2.7. Statistical Analysis

The results were analyzed using both GraphPad Prism 9 and R software programs for statistics and graphical presentation. The following statistics were used to determine the significance of a given observation: For NEC incidence, a Fisher’s exact test was used to assess the differences between the groups; for NEC severity, a one-way ANOVA was used to assess the differences between the groups; for the survival curve analysis, a log-rank test for trend was used; for gross and histological severity scores, Tukey’s multiple comparisons test was performed to assess the differences in the severity score of each section of the GI tract per group. Comparisons of relative abundance of bacterial species across groups and diagnosis were performed using non-parametric Kruskal-Wallis with a Benjamin-Hochberg *p*-value adjustment and Wilcoxon Rank Sum Test, respectively. The codes used for the data analysis and figure generation can be accessed via https://github.com/valeriamelendezhebib/Probiotics-NEC.git (accessed on 12 April 2023). 

## 3. Results

### 3.1. SL and BL. infantis Supplementation Did Not Confer Protection against NEC Incidence or Severity

**Figure 2:**
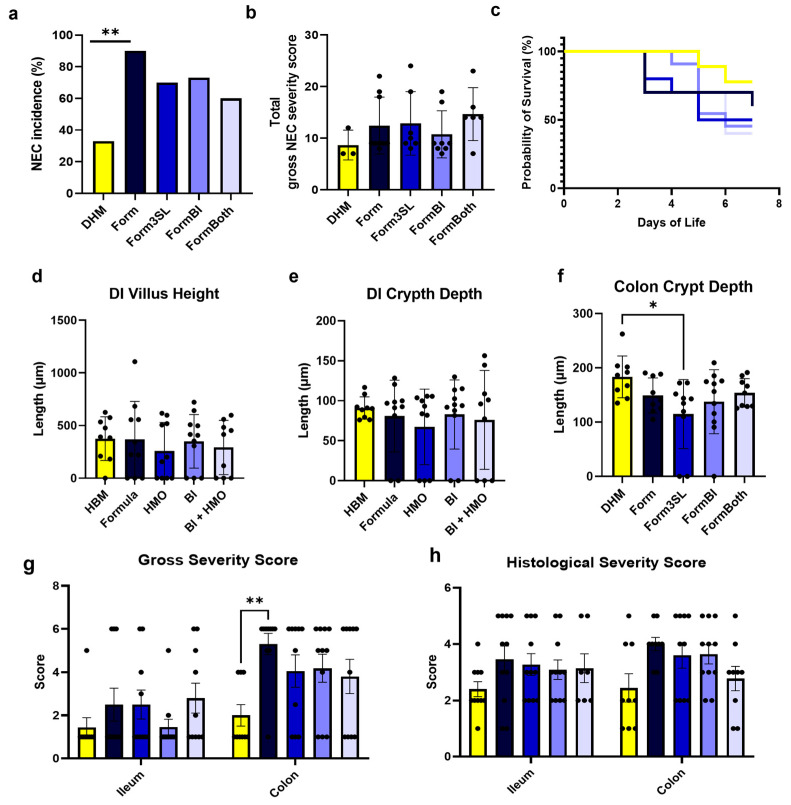
Phenotypic study outcomes. (**a**) NEC incidence per treatment group (%). *p*-value was determined using a Fischer’s Exact Test; **: *p*-value ≤ 0.01 between DHM and Form. (**b**) Total gross NEC severity score. (**c**) Survival curve per treatment group. (**d**) Distal ileum villus height (**e**) Distal ileum crypt depth. (**f**) Colon crypt depth; *p*-value was determined using a Two-way ANOVA, Tukey’s multiple comparisons test; *: *p*-value ≤ 0.05 between DHM and Form3SL. (**g**) Gross severity score of ileum and colon; *p*-value was determined using a Two-way ANOVA, Tukey’s multiple comparisons test; *: *p*-value ≤ 0.05 between DHM and Form. (**h**) Histological severity score of ileum and colon. DHM = donor human milk (n = 9), Form = Formula (n = 10), Form3SL = Formula + 3′SL (n = 10), FormBI = Formula + *BL. infantis* (n = 11), FormBoth = Formula + *BL. infantis* + 3′S (n = 10).

Piglets fed formula exhibited a higher (*p* ≤ 0.01) incidence of NEC (90%) (9/10 animals) than those fed donor human milk (DHM) (33%) (3/9 animals) (Figure 2a). Supplementing formula with sialyllactose (3′-SL) alone resulted in a NEC incidence of 70% (7/10 animals), whereas supplementation with *BL. infantis* alone resulted in a NEC incidence of 72.7% (8/11 animals). Pigs fed formula supplemented with both 3′SL and *BL. infantis* exhibited a lower NEC incidence of 60% (6/10 animals), but this was not statistically different from formula alone.

**Figure 3:**
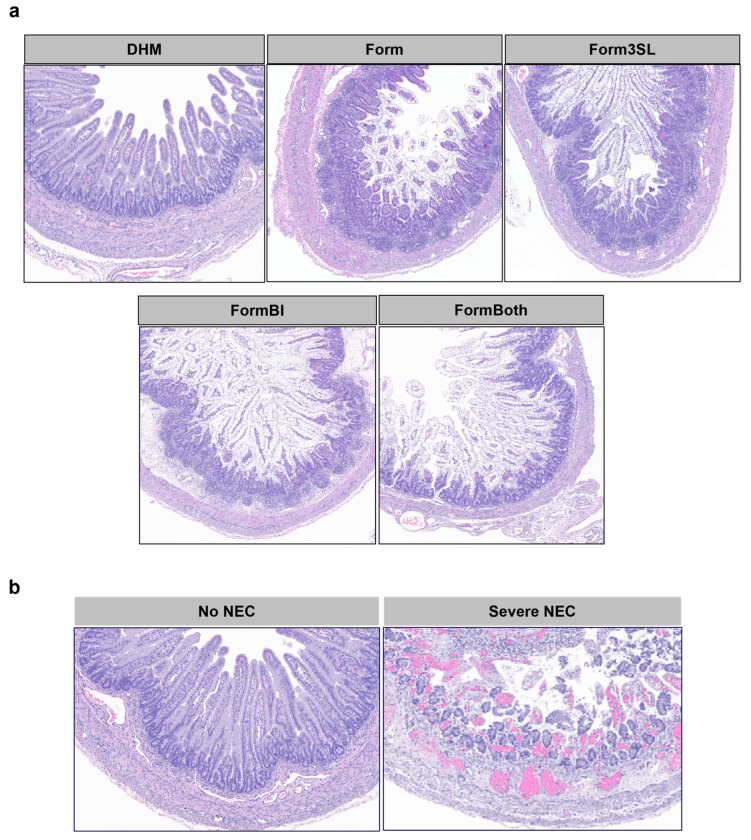
Representative images of distal ileum by treatment group and diagnosis. (**a**) Representative images of distal ileum showing villus and crypt morphology of all treatment groups. (**b**) Representative images of damage to distal ileum in NEC and no NEC. Image illustrating severe NEC represents a tissue severity score of 5.

Measurement of the expression of pro-inflammatory cytokines using distal ileum tissue through qPCR (Supplementary Figure S2a) revealed a significant increase in the expression of IL-1^β^ and TNF-α in NEC compared to healthy animals. Although we observed no significant differences in the expression of all cytokines tested between treatment groups, we observed that the DHM-fed piglets exhibited a lower expression of IL-1β, IL-8, and IL-17A. An assessment of the gross pathology in major GI regions (stomach, jejunum, ileum, and colon) at the time of necropsy revealed that the DHM piglets had a significantly lower NEC severity score than the piglets in the Form group in the colon. There were no other significant differences in the gross NEC severity score between the groups (Figure 2b and Supplementary Figure S1a). A histological assessment of the tissue damage (Figure 2d and Supplementary Figure S1b) in the jejunum, ileum, and colon sections revealed no significant differences in the NEC severity scores. The histological measurements showed similar villus height and crypt depth in the distal ileum of all the animals across the treatments groups (Figure 2d,e). In contrast, a significant increase in the crypt depth of the colon between the DHM- and 3SL-fed piglets (*p* ≤ 0.05) was observed (Figure 2f). The representative images shown in Figure 3a demonstrate the average differences in villus height and crypt depth between all the treatment groups. The images shown in Figure 3b illustrate the difference in tissue damage in the distal ileum between the healthy and severely diseased piglets.

### 3.2. Relative Abundance of Top 10 Taxa by Treatment and Diagnosis 

**Figure 4:**
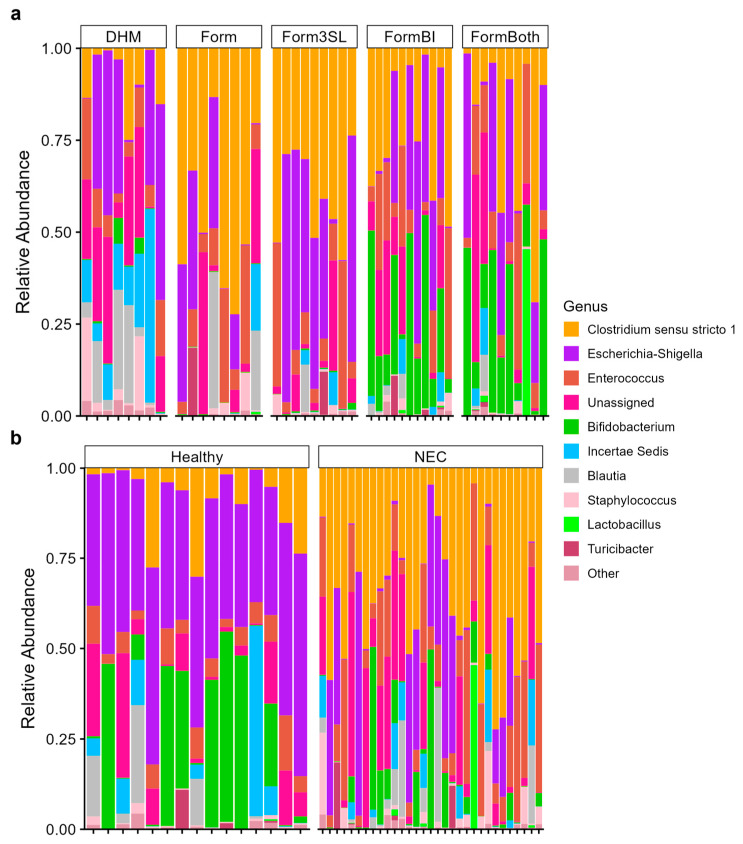
Relative abundance by 16S sequencing of top 10 genera in colon contents. (**a**) Relative abundance of top 10 genera present in colon contents in NEC (n = 31) vs. healthy piglets (n = 15). (**b**) Relative abundance of top 10 genera present in colon contents in all treatment groups (DHM (n = 9), Form (n = 8), Form3SL (n = 9), FormBI (n = 11), and FormBoth (n = 10)).

Analysis of the 16S rRNA gene sequences from the stomach, small intestinal, and colon contents of all the piglets revealed differences in the gut microbiome composition in the piglets by their treatment and diagnosis (Figure 4, Supplementary Figures S4 and S5). In colon contents, the piglets of all the treatment groups exhibited different relative abundances of the top 10 genera present (Figure 4a). Namely, we observed that the DHM-fed piglets possessed an abundance of the genera *Clostridium sensu stricto 1* while all formula-fed piglets (Form, Form3SL, FormBI, and FormBoth) exhibited a higher abundance of this genus. The abundance of *Clostridium sensu stricto 1* seems to decrease when the genus *Bifidobacterium* is introduced into the diet. The comparison between healthy and diseased piglets (Figure 4b) shows a higher relative abundance of the genus Escherichia-Shigella in the healthy piglets, while the NEC-afflicted piglets exhibit a high relative abundance of *Clostridium sensu stricto 1* and *Enterococcus* genera. Similar patterns in relative abundance are also observed in the small intestinal content samples (Supplementary Figure S5). In the stomach contents, we observed a higher relative abundance of *Escherichia-Shigella* in healthy piglets, while we did not observe any striking differences in the abundance of the top 10 genera between the treatment groups (Supplementary Figure S4).

**Figure 5:**
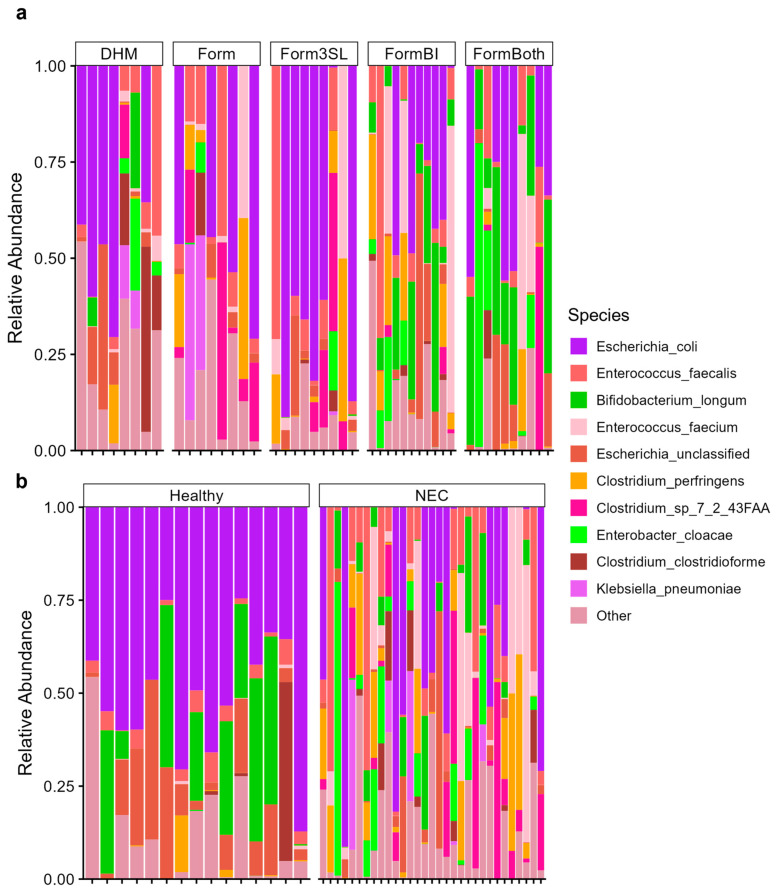
Relative abundance by WGS of top 10 genera in colon contents. (**a**) Relative abundance of top 10 genera present in small intestinal contents in NEC (n = 31) vs. healthy piglets (n = 15). (**b**) Relative abundance of top 10 genera present in small intestinal contents in all treatment groups (DHM (n = 9), Form (n = 8), Form3SL (n = 9), FormBI (n = 11), and FormBoth (n = 10)).

Analysis of the gut microbiome through whole-genome sequencing (WGS) of the colon contents to obtain species resolution revealed that the healthy animals possessed a high abundance of *Escherichia coli* (*E. coli*) and an unclassified Escherichia, while NEC-afflicted animals exhibited a higher abundance of Enterococcus faecalis, Clostridium perfringens, and Clostridium_so_7_2_43FAA (Figure 5a). WGS confirmed the presence of the probiotic Bifidobacterium longum in the FormBI and FormBoth groups, while revealing no other striking differences in the abundance of the top 10 most abundant species between the treatment groups (Figure 5a).

### 3.3. Bifidobacterium Genus and B. longum Are Significantly More Abundant in FormBI and FormBoth Groups and Negatively Correlate with Disease Severity 

**Figure 6:**
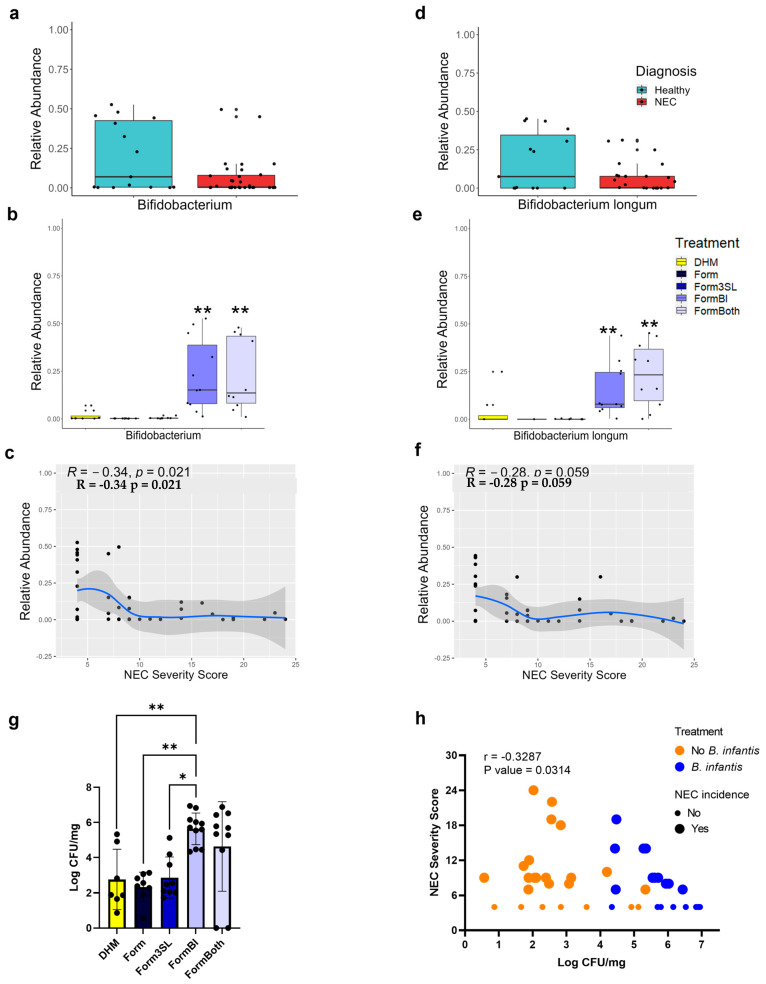
*Bifidobacterium longum* abundance dynamics and correlation with disease severity in colon contents through whole-genome sequencing (WGS), 16S sequencing, and qPCR. (**a**) Relative abundance of *Bifidobacterium* genus in colon contents in NEC vs. Healthy by 16S sequencing. (**b**) Relative abundance of *Bifidobacterium* genus in colon contents in all treatment groups by 16S sequencing; *p*-value was determined using Kruskal-Wallis multiple comparisons followed by *p*-value adjustment with the Benjamin-Hochberg method; **: *p*-value ≤ 0.01; *: *p*-value ≤ 0.05 FormBI and FormBoth vs. all other treatments (**b**). (**c**) Spearman correlation of the relative abundance of *Bifidobacterium* in colon contents and total NEC severity score. (**d**) Relative abundance of *Bifidobacterium longum* species in colon contents in NEC vs. Healthy by WGS. (**e**) Relative abundance of *Bifidobacterium longum* species in colon contents in all treatment groups by WGS; *p*-value was determined using Kruskal-Wallis multiple comparisons followed by *p*-value adjustment with the Benjamin-Hochberg method; **: *p*-value ≤ 0.01; *: *p*-value ≤ 0.05 FormBI and FormBoth vs. all other treatments (**e**). (**f**) Spearman correlation of the relative abundance of *Bifidobacterium longum* species in colon contents and total NEC severity score. (**g**) Log CFU/mg of *B. longum* in NEC vs. healthy piglets in colon contents measure by qPCR; *p*-value was determined by two-way ANOVA **: *p*-value ≤ 0.01; *: *p*-value ≤ 0.05. (**h**) Spearman correlation of Log CFU/mg of *B. longum* in colon contents and NEC severity score by qPCR. Orange represents piglets in groups FormBI and FormBoth. Blue represents piglets in groups DHM, Form, and Form3SL. For panels (**a**) and (**d**), NEC (n = 31) and Healthy (n = 15). For panels (**b**), (**e**) and (**g**), DHM (n = 9), Form (n = 8), Form3SL (n = 9), FormBI (n = 11), and FormBoth (n = 10). For panels (**c**) and (**f**), n = 46. For panels (**h**), no *B. infantis* (n = 25), *B. infantis* (n = 22).

Sequencing of the stomach, small intestine, and colon contents showed that the piglets fed the probiotic *BL. infantis* (animals in the FormBI and FormBoth groups) had significantly (*p* ≤ 0.01) more *Bifidobacterium* than the remaining groups (Figure 6b and Supplementary Figures S6b and S7b). There was no increase in the abundance of *Bifidobacterium* in the piglets that received both *BL. infantis* and 3′SL compared to the piglets that received only *BL. infantis* in any of the intestinal segments.

WGS of colon contents revealed that the relative abundance of *B. longum* is significantly higher in the FormBI and FormBoth groups, consistent with probiotic administration (Figure 6e). Both 16S sequencing and WGS revealed that the abundance of *Bifidobacterium* and *B. longum*, respectively, were modestly negatively correlated with disease severity in the colon (Figure 6c,F), small intestine (Supplementary Figure S7c), and stomach (Supplementary Figure S6c). A quantitative real-time PCR of the colon contents, used to confirm the presence of *B. longum*, demonstrated that the piglets in the FormBI group had significantly (*p* < 0.05) greater levels of *B. longum* compared to the piglets that were not given the probiotic (Figure 6g). The level of *B. longum* in the FormBoth group was higher but was not significantly different from either the FormBI or the other untreated groups. The correlation between the Log CFU/mg and total NEC severity score revealed a significant negative correlation between the abundance of the probiotic and disease severity (Figure 6h).

### 3.4. Escherichia-Shigella Is Significantly More Abundant in Healthy Piglets and Negatively Correlates with Disease Severity

**Figure 7:**
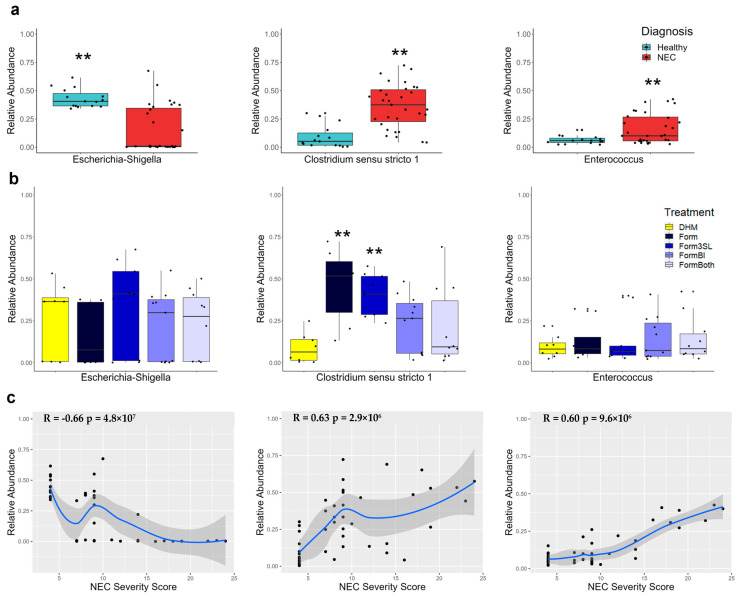
Relative abundance and correlation with disease severity of top 3 most abundant genera in colon contents by 16S sequencing. (**a**) Relative abundance of *Escherichia*-*Shigella*, *Clostridium sensu stricto 1*, and *Enterococcus* genera in colon contents by diagnosis; *p*-value was determined using a Wilcoxon rank sum exact test; **: *p*-value ≤ 0.01 healthy (n = 15) vs. NEC (n = 31) (**a**). (**b**) Relative abundance of Escherichia-Shigella, Clostridium sensu stricto 1, and Enterococcus genera in colon contents by treatment group; *p*-value was determined using Kruskal-Wallis multiple comparisons followed by *p*-value adjustment with the Benjamin-Hochberg method; **: *p*-value ≤ 0.01 Form and Form3SL vs. DHM (**b**). (DHM (n = 9), Form (n = 8), Form3SL (n = 9), FormBI (n = 11), and FormBoth (n = 10)). (**c**) Spearman correlation of relative abundance of *Escherichia*-*Shigella*, *Clostridium sensu stricto 1*, and *Enterococcus* with total NEC severity score (n = 46).

Analysis of 16S rRNA gene sequences from the stomach (*p* ≤ 0.01), small intestinal (*p* ≤ 0.01), and colon (*p* ≤ 0.01) contents showed a significantly higher relative abundance of the genus *Escherichia-Shigella* in the healthy piglets compared to the piglets with NEC (Figure 7a and Supplementary Figures S6a and S7a). A Spearman correlation between the relative abundance of *Escherichia-Shigella* and NEC severity score in all the piglets and revealed a significant negative correlation in all three tissue sites (Figure 7c and Supplementary Figures S6c and S7c). No significant differences in relative abundance of this genus were observed between the treatment groups (Figure 7b and S6b and S7b).

### 3.5. Clostridium sensu stricto 1 Is Significantly More Abundant in Diseased Piglets and Correlates with Disease Severity

Analysis of 16S rRNA gene sequences from the stomach (*p* ≤ 0.01), small intestinal (*p* ≤ 0.01), and colon (*p* ≤ 0.01) contents showed a significantly higher relative abundance of the genus *Clostridium sensu stricto 1* in the piglets that developed NEC compared to the healthy piglets (Figure 7a and Supplementary Figures S6a and S7a). A Spearman correlation between the relative abundance of *Clostridium sensu stricto 1* and NEC severity score in all the piglets revealed a significant positive correlation in all three tissue sites (Figure 7c and Supplementary Figures S6c and S7c). 

### 3.6. Enterococcus Is Significantly More Abundant in the Colon of Diseased Piglets and Correlates with Disease Severity

Analysis of 16S rRNA gene sequences from the stomach, small intestinal, and colon (*p* ≤ 0.01) contents showed a significantly higher relative abundance of the genus *Enterococcus* in the colons of the piglets that developed NEC compared to the healthy piglets (Figure 7a). No significant difference in the abundance of this genus through diagnosis was observed in the small intestine (Supplementary Figure S7a). No significant differences were found between the treatment groups in all the segments of the intestine (Figure 7b and Supplementary Figures S6b and S7b). A Spearman correlation between the relative abundance of *Enterococcus* and NEC severity score in all the piglets revealed a significant positive correlation in both the small intestine and colon (Figure 7c and Supplementary Figure S7c). 

### 3.7. Donor Human Milk-Fed Piglets Exhibited Lower Abundance of Clostridium sensu stricto 1

Analysis of 16S rRNA gene sequences from the small intestine and colon contents also revealed that the DHM-fed piglets had significantly lower levels of *Clostridium sensu stricto 1* in the colon compared to the formula fed piglets that were not fed *BL. infantis*, Form (*p* ≤ 0.01) and Form3SL (*p* ≤ 0.01) (Figure 7b).

### 3.8. Clostridium Perfringens Is Significantly More Abundant in Piglets with NEC and Positively Correlates with Disease Severity

**Figure 8:**
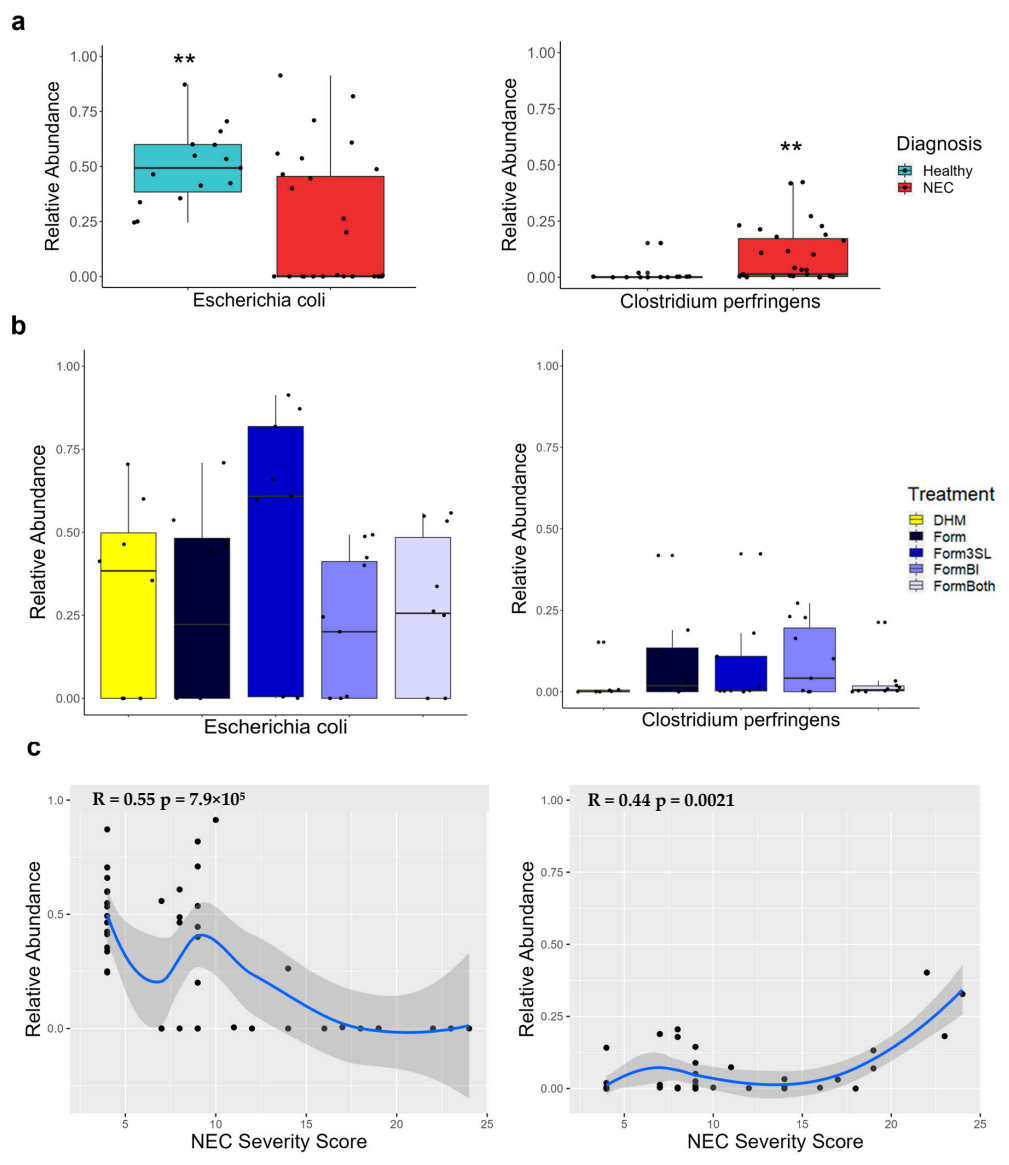
Relative abundance and correlation with disease severity of *Escherichia coli* and *Clostridium perfringens* in colon contents through whole-genome sequencing (WGS). (**a**) Relative abundance of *Escherichia coli* (*E. coli*) and *Clostridium perfringens* in NEC (n = 31) vs. healthy (n = 15) piglets. *p*-value was determined using a Wilcoxon rank sum exact test, ** *p*-value ≤ 0.01 healthy vs. NEC. (**b**) Relative abundance of *E. coli* and *C. perfringens* in all treatment groups. (DHM (n = 9), Form (n = 8), Form3SL (n = 9), FormBI (n = 11), and FormBoth (n = 10)), (**c**) Spearman correlation of the relative abundance of *E. coli* and *C. perfringens* with total NEC severity score (n = 46).

Whole-genome sequencing of the colon contents revealed that *Clostridium perfringens* (*C. perfringens*) species was significantly more abundant in the piglets with NEC than in the healthy piglets (*p* ≤ 0.01) (Figure 8a). However, there were no significant (*p* > 0.05) differences in the abundance of *C. perfringens* between the treatment groups (Figure 8d). A Spearman correlation revealed that the abundance of *C. perfringens* was significantly positively correlated with disease severity (Figure 8c).

### 3.9. Escherichia coli Is Significantly More Abundant in Healthy Piglets and Negatively Correlates with Disease Severity

Whole-genome sequencing of the colon contents revealed that *E. coli* species were significantly more abundant in the healthy piglets than in the NEC piglets (*p* ≤ 0.01) (Figure 8a). There were no significant differences in the abundance of this species between the treatment groups (*p* ≤ 0.01) (Figure 8n). A Spearman correlation revealed that the abundance of *E. coli* was negatively correlated with disease severity (Figure 8c). 

### 3.10. 16S Sequencing Shows Daily Patterns of Abundance of the Top 5 Genera in the Stool

**Figure 9:**
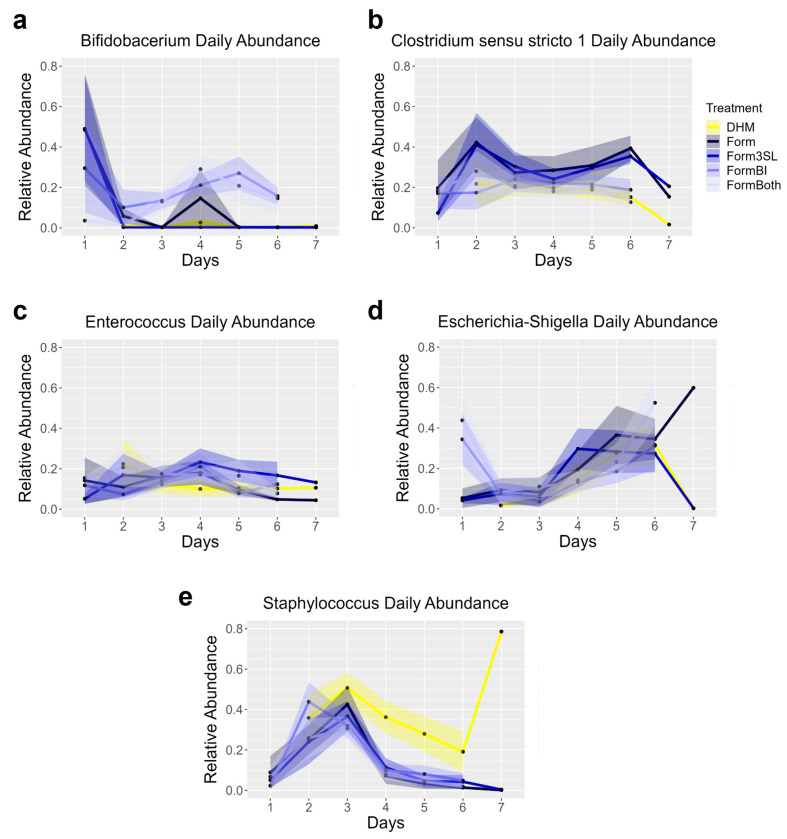
Daily relative abundance of top 5 taxa in stool by 16S sequencing. (**a**) Daily abundance of *Bifidobacterium* in all treatment groups. (**b**) Daily abundance of *Clostridium sensu stricto 1* in all treatment groups. (**c**) Daily abundance of *Enterococcus* in all treatment groups. (**d**) Daily abundance of *Escherichia*-*Shigella* in all treatment groups. (**e**) Daily abundance of *Staphylococcus* in all treatment groups. Data points represent mean relative abundance of all piglets of the same group. Shaded area represents standard error of the mean. For all panels, DHM (n = 9), Form (n = 8), Form3SL (n = 9), FormBI (n = 11), and FormBoth (n = 10).

Sequencing of the daily rectal stool samples collected from all the piglets revealed that the most abundant genera in the stool were *Bifidobacterium*, *Clostridium sensu stricto 1*, *Enterococcus*, *Escherichia-Shigella*, and *Staphylococcus* (Figure 9). The piglets from the Form, Form3SL, and FormBI groups exhibited a high relative abundance of a *Bifidobacterium* at day 1, before probiotic supplementation began (Figure 9a).

## 4. Discussion

In this study, we used a clinically relevant animal model to evaluate not only the effectiveness of a pre- and probiotic treatment in reducing the incidence of NEC, but also how these interventions shape the gut microbiota composition under controlled feeding regimens. Several clinical studies have reported that the use of *BL. infantis* alone or within probiotic mixtures, correlates with reductions in NEC incidence and gastrointestinal morbidity [19,20,21,22]. Interpreting the results of most infant studies is complicated by the differing probiotic mixtures and feeding regimes used. Therefore, we sought to evaluate the effectiveness of *BL. infantis* as a singular probiotic in infant formula-fed preterm piglets since this diet is associated with the greatest risk for NEC. The preterm piglets used in this study reflect the stage of gastrointestinal development of an infant born at 28–30 weeks gestation [30], a very at-risk population for NEC. Aside from the metabolic, nutritional, and immunological similarities, the preterm piglet spontaneously develops NEC when fed a commercially available infant formula, but not human milk, much like the preterm infant [31,32,33,34,35]. We also employed repeated fecal sampling and dual sequencing approaches to more rigorously and comprehensively characterize the microbiota and assess how well the preterm piglet models the human preterm infant. Here, we confirmed previous findings that donor human milk (DHM) confers protection against NEC in piglets [35], as it does in preterm infants [2,3]. We also found that supplementing *BL. infantis*, alone or together with 3′SL, increased the abundance of *B. longum*, which is believed to represent *BL. infantis* in this study, and this was correlated with lower disease severity in the colons of the formula-fed piglets. 

Our results show that supplementing preterm infant formula with *BL. infantis*, with and without 3′SL, modestly decreased the severity of NEC. We found that the abundance of *B. longum* was marginally higher in the healthy piglets. Human infant studies have reported a wide range of probiotic dosages (CFU/day), and it is not currently known if there is an optimal number of bacteria needed to obtain maximum benefits from probiotic species. It is possible that a higher dose of *BL. infantis* would yield definitive protective effects against NEC in this model. However, as previously mentioned, it is difficult to ascertain, based on human infant studies, if the beneficial effects of *BL. infantis* administration stem from the probiotic alone or are aided by confounding factors such as the feeding of human milk which is known to significantly decrease the risk of NEC.

We show that the provision of 3′SL as a representative HMO does not increase the abundance of *B. longum* or its ability to prevent NEC. This suggests that this particular HMO alone was not sufficient to increase the effectiveness of *BL. infantis*. Both 3′SL and *BL. infantis* have been shown to have beneficial effects on the host [36]. Administration of 3′SL alone has been reported to exert remarkable anti-inflammatory effects on the host and can even confer protection against a pathogenic infection in vitro [37,38,39,40,41]. It is possible the *B infantis* feeding or its combination with a single HMO were not sufficient to combat the detrimental effects of exclusive formula feeding and prematurity of the intestinal barrier and the immune system [31]. It was notable that we did not find a significant increase in the abundance of bifidobacteria in the DHM-fed piglets, despite the presence of a more complex HMO profile. This could be because the DHM used in this study was pasteurized and thus did not contain any live bifidobacteria. Our results suggest that a single HMO fed with formula or pasteurized DHM feeding alone may not be a sufficient dietary approach to enable robust colonization with bifidobacteria. 

We sought to examine whether the microbiota community composition measured in our preterm pigs was similar to that reported in human preterm infants using rigorous sampling and analytical approaches. This was also unique in that it enabled us to characterize the evolution of the bacterial community in cesarean-derived, preterm piglet in the first week after birth. It was evident from the daily 16S rRNA gene sequencing of the rectal stool samples that there was a relatively small number of dominant bacterial taxa in the first week of life. Several of the dominant bacterial genera (*Escherichia-Shigella*, *Clostridium sensu stricto 1*, *Enterococcus*, *Bifidobacterium*, and *Staphylcoccus*) and species (*E. coli*, *C. perfringens*, *Klebsiella pneumoniae*, *B. longum*, and *Enterococcus faecium*) that we measured in our piglet rectal samples and small intestine and colon contents have been reported in various human preterm infant studies [1,6,9,42,43]. This finding was reassuring in the context of how well the preterm piglet models the human preterm infant. However, it raises the important question of the source of the bacteria that colonize the newborn gut, especially in conditions of cesarean-delivery. It also highlights the opportunity to use therapeutic intervention to shape what seems to be a simple microbial community at birth. 

We have previously reported discrepancies in the gut microbiota composition of healthy and diseased piglets and differences in diet composition [24]. The discrepancies in the gut microbiota reported in healthy infants and those with NEC have provided insight into how various bacteria may contribute to the pathogenesis of NEC [6,8]. Here, we observed remarkable differences in the relative abundance of *Clostridium sensu stricto 1*. The piglets that developed NEC had a significantly higher abundance of this genus, and this abundance exhibited strong positive correlations with disease severity. Importantly, DHM-fed piglets had a marked reduction in the abundance of this taxa. This suggests that diet can influence the microbial composition in significant ways and that exclusive formula-based feeding may lead to the growth of these potentially harmful bacteria. Moreover, we observed no significant differences in the abundance of *Clostridium sensu stricto 1* between the groups fed *BL. infantis* and the DHM-fed pigs. This finding suggests that probiotics have the potential to decrease colonization by other bacterial taxa that we found to be associated with NEC. Human milk possesses a plethora of bioactive molecules that are thought to, among many other things, shape the gut microbiome, aid in the maturation of the intestinal epithelium, and possess antimicrobial properties [44]. Our results suggest that one of the ways that human milk protects against NEC is by modulating the gut microbiota. The mechanisms through which it does so warrant further study.

*Clostridium* species are often found to colonize the gut of preterm infants and are capable of inflicting substantial damage to the gastrointestinal epithelium and cause diseases like enteritis and enterotoxemia through the production of harmful toxins [45]. Moreover, *Clostridium* spp. have been implicated in NEC based on culture and sequence-based analyses of infant stool samples [46,47,48,49]. *Clostridium perfringens* (*C. perfringens*), a notable member of the *Clostridium sensu stricto 1* genus capable of inflicting such diseases, was identified in this study through whole-genome sequencing (WGS) of colon contents. Our analysis showed that *C. perfringens*, like *Clostridium sensu stricto 1*, was present in significantly higher levels in the piglets with NEC and correlated with disease severity. Daily sampling of the stool microbiome showed that the relative abundance of *Clostridium sensu stricto 1* was at its highest starting at day 2 of life and remained relatively stable throughout the duration of the study. This suggests that colonization with *Clostridium sensu stricto 1* begins very early after birth in preterm piglets and may precede and contribute to the pathogenesis of NEC, which has been observed in infant studies [46]. It is notable that a number of human clinical studies have implicated other bacterial taxa in the pathogenesis of NEC, namely *Enterobacteriaceae*, which includes species such as *Klebsiella* and *E. coli* [6,8]. Consistent with studies on humans, we found that *Escherichia-Shigella*, specifically *E. coli*, was abundant in the small intestine and colon in our preterm piglets, but surprisingly the relative abundance was higher in the healthy piglets than in those that developed NEC, and it was negatively correlated with NEC severity, suggesting the specific strains present were not pathogenic.

## 5. Conclusions

Our results show that the administration of *BL. infantis* with and without 3′SL resulted in a marginal reduction in the incidence of NEC in a preterm piglet model but did not achieve the protective effects observed with DHM. We showed that the intestinal abundance of *B. longum* negatively correlated with disease severity, suggesting that although it did not yield robust protection, it might exert beneficial effects in the host, even when fed in the context of exclusive formula feeding. We also showed that the gut microbiota composition in preterm piglets has similarities to that reported in human preterm infants and revealed novel insights into the possible links between NEC severity and specific taxa, namely *Clostridium perfringens*. Our findings also point to important questions about how diet composition shapes the structure of the gut microbiota and the risk for NEC.

## Figures and Tables

**Table 1:** Forward and reverse primers used for quantification of *B. longum*.

Direction	Organism	Taxa Level	Gene Target	Amplicon Size	Sequence (5′-3′)
Forward	Bacteria	*B. longum* group	16S rRNA	106	CAGTTGATCGCATGGTCTT
Reverse	Bacteria	*B. longum* group	16S rRNA	106	TACCCGTCGAAGCCAC

## Data Availability

Raw data files have been submitted to the NCBI BioProject data base under accession number PRJNA948045. Codes used for data analysis can be accessed through https://github.com/valeriamelendezhebib/Probiotics-NEC.git (accessed on 12 April 2023).

## Supplementary Materials

The supporting information can be downloaded at: https://www.mdpi.com/article/10.3390/nu15112585/s1.

## References

[1] Thanert R., Keen E.C., Dantas G., Warner B.B., Tarr P.I. (2021). Necrotizing Enterocolitis and the Microbiome: Current Status and Future Directions. J. Infect. Dis..

[2] Lucas A., Cole T.J. (1990). Breast milk and neonatal necrotising enterocolitis. Lancet.

[3] Meinzen-Derr J., Poindexter B., Wrage L., Morrow A.L., Stoll B., Donovan E.F. (2009). Role of human milk in extremely low birth weight infants’ risk of necrotizing enterocolitis or death. J. Perinatol..

[4] Rose A.T., Patel R.M. (2018). A critical analysis of risk factors for necrotizing enterocolitis. Semin. Fetal. Neonatal Med..

[5] Jensen M.L., Thymann T., Cilieborg M.S., Lykke M., Molbak L., Jensen B.B., Schmidt M., Kelly D., Mulder I., Burrin D.G. (2014). Antibiotics modulate intestinal immunity and prevent necrotizing enterocolitis in preterm neonatal piglets. Am. J. Physiol. Gastrointest. Liver Physiol..

[6] Warner B.B., Deych E., Zhou Y., Hall-Moore C., Weinstock G.M., Sodergren E., Shaikh N., Hoffmann J.A., Linneman L.A., Hamvas A. (2016). Gut bacteria dysbiosis and necrotising enterocolitis in very low birthweight infants: A prospective case-control study. Lancet.

[7] Sangild P.T., Siggers R.H., Schmidt M., Elnif J., Bjornvad C.R., Thymann T., Grondahl M.L., Hansen A.K., Jensen S.K., Boye M. (2006). Diet- and colonization-dependent intestinal dysfunction predisposes to necrotizing enterocolitis in preterm pigs. Gastroenterology.

[8] Gopalakrishna K.P., Macadangdang B.R., Rogers M.B., Tometich J.T., Firek B.A., Baker R., Ji J., Burr A.H.P., Ma C., Good M. (2019). Maternal IgA protects against the development of necrotizing enterocolitis in preterm infants. Nat. Med..

[9] Heida F.H., van Zoonen A., Hulscher J.B.F., Te Kiefte B.J.C., Wessels R., Kooi E.M.W., Bos A.F., Harmsen H.J.M., de Goffau M.C. (2016). A Necrotizing Enterocolitis-Associated Gut Microbiota Is Present in the Meconium: Results of a Prospective Study. Clin. Infect. Dis..

[10] Kiu R., Shaw A., Sim K., Bedwell H., Cornwell E., Pickard D., Belteki G., Malsom J., Philips S., Young G.R. (2021). Dissemination and pathogenesis of toxigenix Clostridium perfringens strains linked to neonatal intensive care units and Necrotising Enterocolitis. bioRxiv.

[11] Autran C.A., Kellman B.P., Kim J.H., Asztalos E., Blood A.B., Spence E.C.H., Patel A.L., Hou J., Lewis N.E., Bode L. (2018). Human milk oligosaccharide composition predicts risk of necrotising enterocolitis in preterm infants. Gut.

[12] Wejryd E., Marti M., Marchini G., Werme A., Jonsson B., Landberg E., Abrahamsson T.R. (2018). Low Diversity of Human Milk Oligosaccharides is Associated with Necrotising Enterocolitis in Extremely Low Birth Weight Infants. Nutrients.

[13] Preidis G.A., Weizman A.V., Kashyap P.C., Morgan R.L. (2020). AGA Technical Review on the Role of Probiotics in the Management of Gastrointestinal Disorders. Gastroenterology.

[14] Autran C.A., Schoterman M.H., Jantscher-Krenn E., Kamerling J.P., Bode L. (2016). Sialylated galacto-oligosaccharides and 2′-fucosyllactose reduce necrotising enterocolitis in neonatal rats. Br. J. Nutr..

[15] Jantscher-Krenn E., Zherebtsov M., Nissan C., Goth K., Guner Y.S., Naidu N., Choudhury B., Grishin A.V., Ford H.R., Bode L. (2012). The human milk oligosaccharide disialyllacto-N-tetraose prevents necrotising enterocolitis in neonatal rats. Gut.

[16] Cilieborg M.S., Sangild P.T., Jensen M.L., Ostergaard M.V., Christensen L., Rasmussen S.O., Morbak A.L., Jorgensen C.B., Bering S.B. (2017). alpha1,2-Fucosyllactose Does Not Improve Intestinal Function or Prevent Escherichia coli F18 Diarrhea in Newborn Pigs. J. Pediatr. Gastroenterol. Nutr..

[17] Rasmussen S.O., Martin L., Ostergaard M.V., Rudloff S., Roggenbuck M., Nguyen D.N., Sangild P.T., Bering S.B. (2017). Human milk oligosaccharide effects on intestinal function and inflammation after preterm birth in pigs. J. Nutr. Biochem..

[18] Underwood M.A. (2022). Bifidobacterium infantis, Necrotizing Enterocolitis, Death, and the Role of Parents in the Neonatal Intensive Care Unit. J. Pediatr..

[19] Hoyos A.B. (1999). Reduced incidence of necrotizing enterocolitis associated with enteral administration of Lactobacillus acidophilus and Bifidobacterium infantis to neonates in an intensive care unit. Int. J. Infect. Dis..

[20] Robertson C., Savva G.M., Clapuci R., Jones J., Maimouni H., Brown E., Minocha A., Hall L.J., Clarke P. (2020). Incidence of necrotising enterocolitis before and after introducing routine prophylactic Lactobacillus and Bifidobacterium probiotics. Arch. Dis. Child Fetal. Neonatal Ed..

[21] Hartel C., Pagel J., Rupp J., Bendiks M., Guthmann F., Rieger-Fackeldey E., Heckmann M., Franz A., Schiffmann J.H., Zimmermann B. (2014). Prophylactic use of Lactobacillus acidophilus/Bifidobacterium infantis probiotics and outcome in very low birth weight infants. J. Pediatr..

[22] Tobias J., Olyaei A., Laraway B., Jordan B.K., Dickinson S.L., Golzarri-Arroyo L., Fialkowski E., Owora A., Scottoline B. (2022). Bifidobacteriumlongum subsp. infantis EVC001 Administration Is Associated with a Significant Reduction in the Incidence of Necrotizing Enterocolitis in Very Low Birth Weight Infants. J. Pediatr..

[23] Ghoneim N., Bauchart-Thevret C., Oosterloo B., Stoll B., Kulkarni M., de Pipaon M.S., Zamora I.J., Olutoye O.O., Berg B., Wittke A. (2014). Delayed initiation but not gradual advancement of enteral formula feeding reduces the incidence of necrotizing enterocolitis (NEC) in preterm pigs. PLoS ONE.

[24] Call L., Stoll B., Oosterloo B., Ajami N., Sheikh F., Wittke A., Waworuntu R., Berg B., Petrosino J., Olutoye O. (2018). Metabolomic signatures distinguish the impact of formula carbohydrates on disease outcome in a preterm piglet model of NEC. Microbiome.

[25] Huda M.N., Lewis Z., Kalanetra K.M., Rashid M., Ahmad S.M., Raqib R., Qadri F., Underwood M.A., Mills D.A., Stephensen C.B. (2014). Stool microbiota and vaccine responses of infants. Pediatrics.

[26] Callahan B.J., McMurdie P.J., Rosen M.J., Han A.W., Johnson A.J., Holmes S.P. (2016). DADA2: High-resolution sample inference from Illumina amplicon data. Nat. Methods.

[27] Bolyen E., Rideout J.R., Dillon M.R., Bokulich N.A., Abnet C.C., Al-Ghalith G.A., Alexander H., Alm E.J., Arumugam M., Asnicar F. (2019). Author Correction: Reproducible, interactive, scalable and extensible microbiome data science using QIIME 2. Nat. Biotechnol..

[28] Liu J., Taft D.H., Maldonado-Gomez M.X., Johnson D., Treiber M.L., Lemay D.G., DePeters E.J., Mills D.A. (2019). The fecal resistome of dairy cattle is associated with diet during nursing. Nat. Commun..

[29] Truong D.T., Franzosa E.A., Tickle T.L., Scholz M., Weingart G., Pasolli E., Tett A., Huttenhower C., Segata N. (2015). MetaPhlAn2 for enhanced metagenomic taxonomic profiling. Nat. Methods.

[30] Burrin D., Sangild P.T., Stoll B., Thymann T., Buddington R., Marini J., Olutoye O., Shulman R.J. (2020). Translational Advances in Pediatric Nutrition and Gastroenterology: New Insights from Pig Models. Annu. Rev. Anim. Biosci..

[31] Oosterloo B.C., Premkumar M., Stoll B., Olutoye O., Thymann T., Sangild P.T., Burrin D.G. (2014). Dual purpose use of preterm piglets as a model of pediatric GI disease. Vet. Immunol. Immunopathol..

[32] Sangild P.T. (2006). Gut responses to enteral nutrition in preterm infants and animals. Exp. Biol. Med..

[33] Sangild P.T., Thymann T., Schmidt M., Stoll B., Burrin D.G., Buddington R.K. (2013). Invited review: The preterm pig as a model in pediatric gastroenterology. J. Anim. Sci..

[34] Lennon D., Zanganeh T., Borum P.R. (2011). Development of the piglet neonatal intensive care unit for translational research. Lab. Anim..

[35] Jensen M.L., Sangild P.T., Lykke M., Schmidt M., Boye M., Jensen B.B., Thymann T. (2013). Similar efficacy of human banked milk and bovine colostrum to decrease incidence of necrotizing enterocolitis in preterm piglets. Am. J. Physiol. Regul. Integr. Comp. Physiol..

[36] Hill D.R., Chow J.M., Buck R.H. (2021). Multifunctional Benefits of Prevalent HMOs: Implications for Infant Health. Nutrients.

[37] Quinn E.M., Slattery H., Walsh D., Joshi L., Hickey R.M. (2020). Bifidobacterium longum subsp. infantis ATCC 15697 and Goat Milk Oligosaccharides Show Synergism In Vitro as Anti-Infectives against *Campylobacter jejuni*. Foods.

[38] Kang L.J., Oh E., Cho C., Kwon H., Lee C.G., Jeon J., Lee H., Choi S., Han S.J., Nam J. (2020). 3′-Sialyllactose prebiotics prevents skin inflammation via regulatory T cell differentiation in atopic dermatitis mouse models. Sci. Rep..

[39] Kavanaugh D.W., O’Callaghan J., Butto L.F., Slattery H., Lane J., Clyne M., Kane M., Joshi L., Hickey R.M. (2013). Exposure of Bifidobacterium longum subsp. infantis to Milk Oligosaccharides Increases Adhesion to Epithelial Cells and Induces a Substantial Transcriptional Response. PLoS ONE.

[40] Rousseaux A., Brosseau C., Le Gall S., Piloquet H., Barbarot S., Bodinier M. (2021). Human Milk Oligosaccharides: Their Effects on the Host and Their Potential as Therapeutic Agents. Front. Immunol..

[41] Angeloni S., Ridet J.L., Kusy N., Gao H., Crevoisier F., Guinchard S., Kochhar S., Sigrist H., Sprenger N. (2005). Glycoprofiling with micro-arrays of glycoconjugates and lectins. Glycobiology.

[42] Ward D.V., Scholz M., Zolfo M., Taft D.H., Schibler K.R., Tett A., Segata N., Morrow A.L. (2016). Metagenomic Sequencing with Strain-Level Resolution Implicates Uropathogenic *E. coli* in Necrotizing Enterocolitis and Mortality in Preterm Infants. Cell Rep..

[43] Olm M.R., Bhattacharya N., Crits-Christoph A., Firek B.A., Baker R., Song Y.S., Morowitz M.J., Banfield J.F. (2019). Necrotizing enterocolitis is preceded by increased gut bacterial replication, Klebsiella, and fimbriae-encoding bacteria. Sci. Adv..

[44] Gopalakrishna K.P., Hand T.W. (2020). Influence of Maternal Milk on the Neonatal Intestinal Microbiome. Nutrients.

[45] Stevens D.L., Aldape M.J., Bryant A.E. (2012). Life-threatening clostridial infections. Anaerobe.

[46] Sim K., Shaw A.G., Randell P., Cox M.J., McClure Z.E., Li M.S., Haddad M., Langford P.R., Cookson W.O., Moffatt M.F. (2015). Dysbiosis anticipating necrotizing enterocolitis in very premature infants. Clin. Infect. Dis..

[47] Shaw A.G., Cornwell E., Sim K., Thrower H., Scott H., Brown J.C.S., Dixon R.A., Kroll J.S. (2020). Dynamics of toxigenic Clostridium perfringens colonisation in a cohort of prematurely born neonatal infants. BMC Pediatr..

[48] Schonherr-Hellec S., Aires J. (2019). Clostridia and necrotizing enterocolitis in preterm neonates. Anaerobe.

[49] de la Cochetiere M.F., Piloquet H., des Robert C., Darmaun D., Galmiche J.P., Roze J.C. (2004). Early intestinal bacterial colonization and necrotizing enterocolitis in premature infants: The putative role of Clostridium. Pediatr. Res..

