# Supplementary material for: Probiotics and Human Milk Differentially Influence the Gut Microbiome and NEC Incidence in Preterm Pigs
Source: Nutrients. 2023 May 31;15(11):2585. doi: 10.3390/nu15112585 (PMC10255242; doi:10.3390/nu15112585)
Supplement: Supplementary file 1 [file nutrients-15-02585-s001.zip › nutrients-2396964-supplementary.pdf]

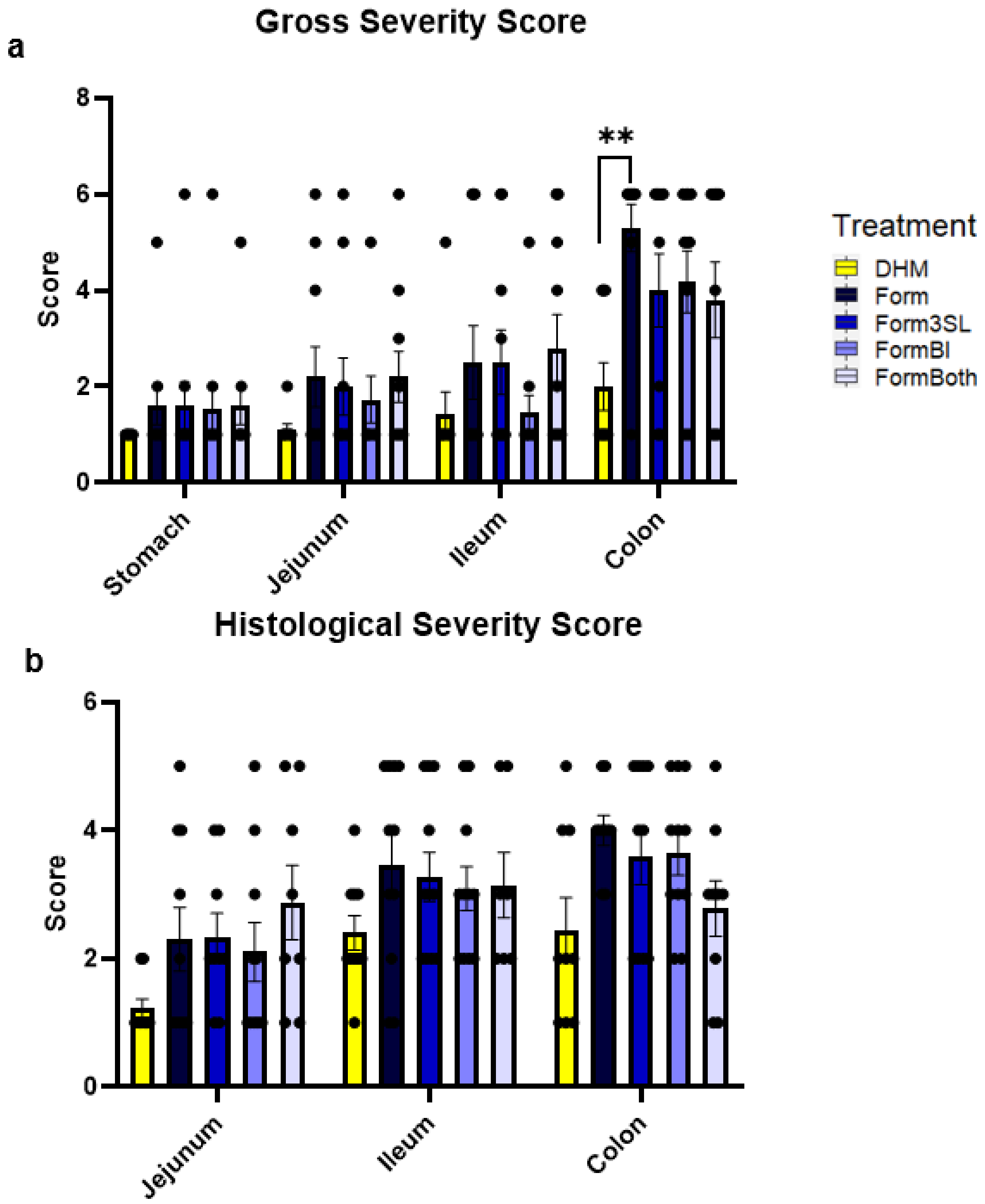

Supplementary Figure S1. Phenotypic study outcomes. (**a**) Gross severity score by intestinal segment. P-value was determined using a Two-way ANOVA, Tukey's multiple comparisons test; **:p-value $\leqslant$ 0.01 between DHM and FormBoth. (**b**) Histological severity score by intestinal segment.

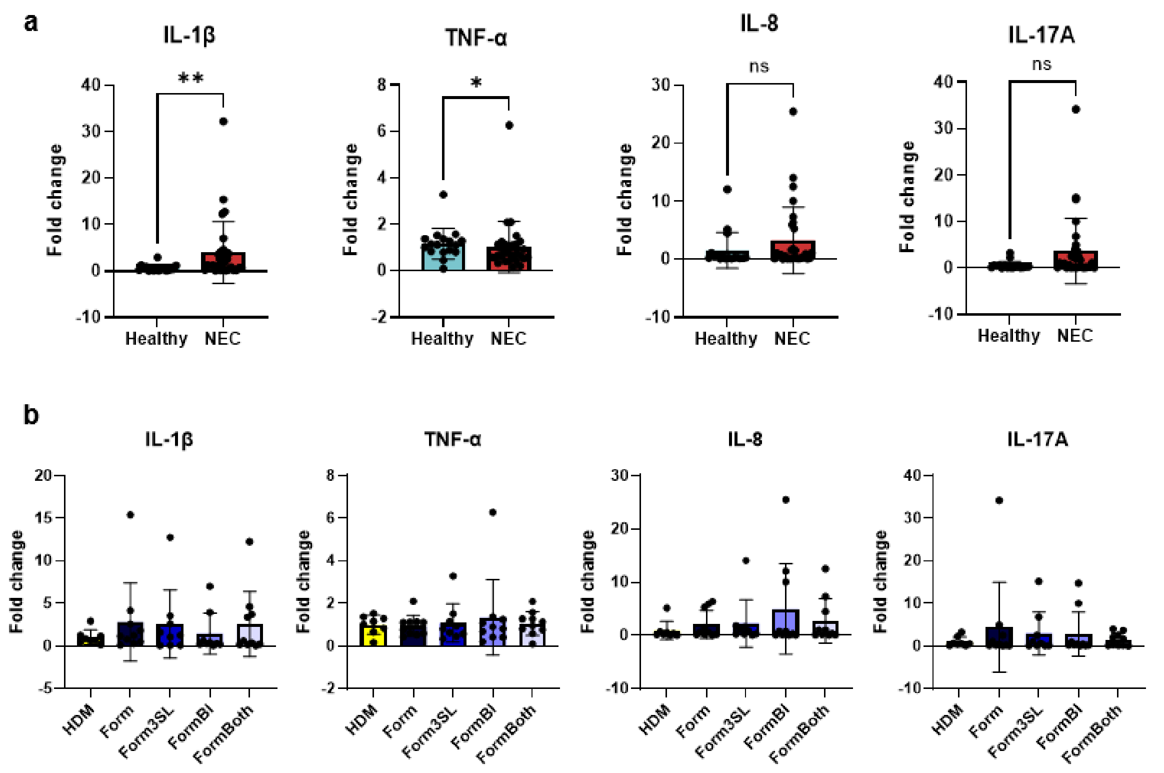

Supplementary Figure S2. Pro-inflammatory cytokine expression analysis in distal ileum tissue. (**a**) Fold change in expression of IL-1$^{\beta}$, TNF-α, IL-8, and IL-7a in healthy vs NEC piglets. (**b**) Fold change in expression of IL-1$^{\beta}$, TNF-α, IL-8, and IL-7a in all treatment groups (DHM (n= 8), Form (n=10), Form3SL (n=9), FormBoth (n=10), FormBI (n=9)).

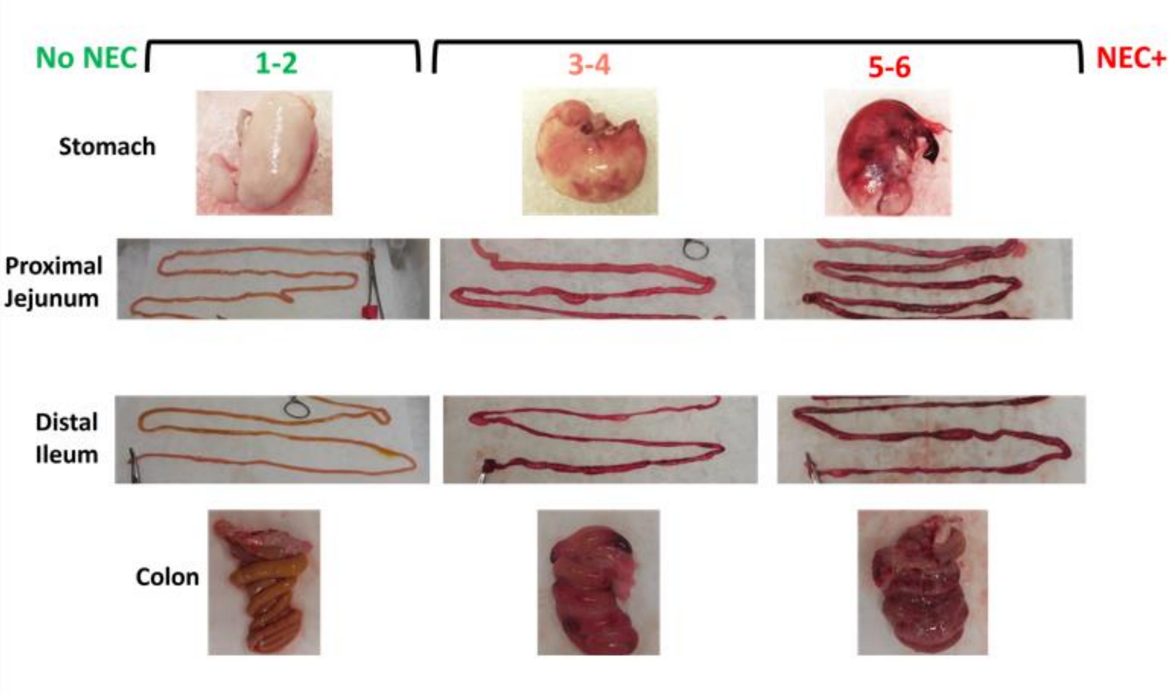

Supplementary Figure S3. Representative images and scoring of gross NEC severity score.

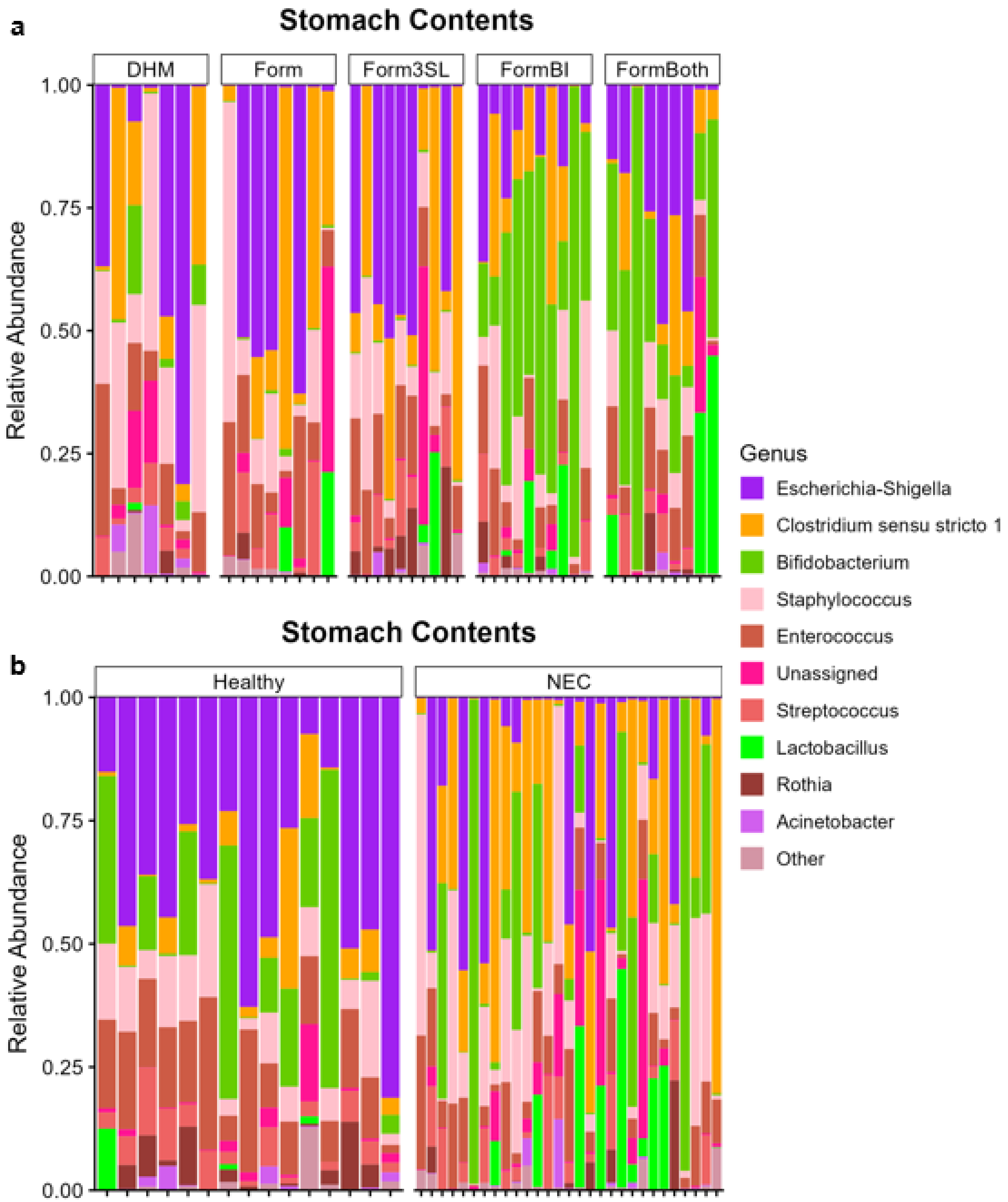

Supplementary Figure S4. Relative abundance by 16S sequencing of top 10 genera in stomach contents. (**a**) Relative abundance of top 10 genera in all treatment groups (DHM (n=9), Form (n=8), Form3SL (n=9), FormBI (n=11), and FormBoth (n=10)). (**b**) Relative abundance of top 10 genera in NEC (n=31) vs healthy (n=15) piglets.

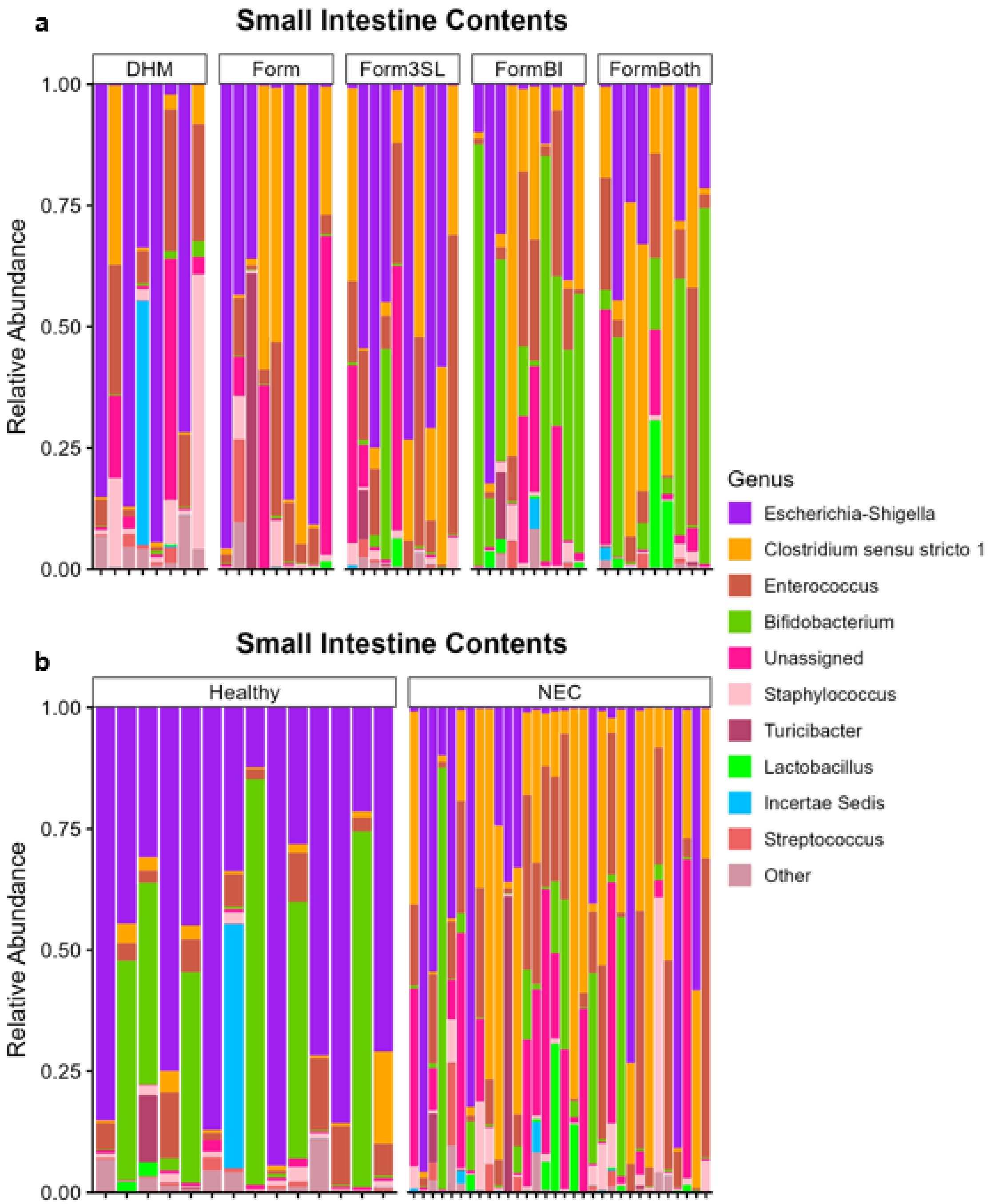

Supplementary Figure S5. Relative abundance by 16S sequencing of top 10 genera in small intestinal contents. (**a**) Relative abundance of top 10 genera present in small intestinal contents in all treatment groups (DHM (n=9), Form (n=8), Form3SL (n=9), FormBI (n=11), and FormBoth (n=10)). (**b**) Relative abundance of top 10 genera present in small intestinal contents in NEC (n=31) vs healthy (n=15) piglets.

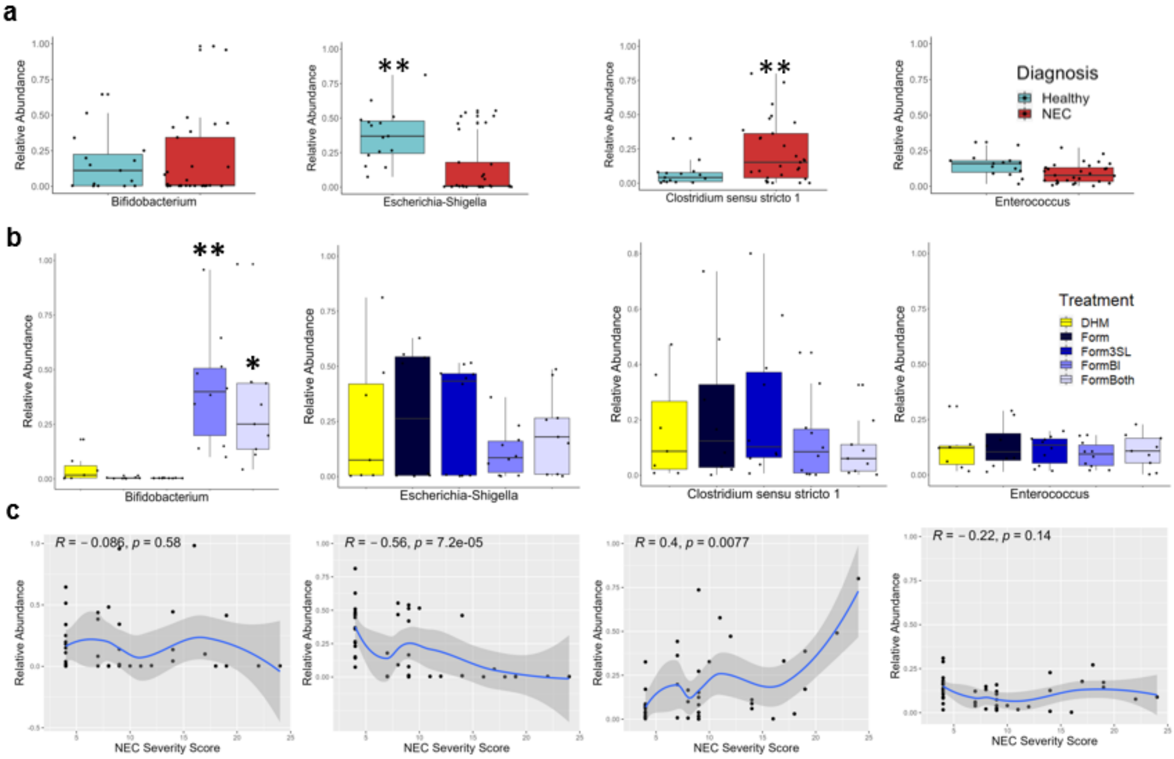

Supplementary Figure S6. Relative abundance and correlation with disease severity of top 3 most abundant genera in stomach contents by 16S sequencing. (**a**) Relative abundance of Bifidobacterium, Escherichia-Shigella, Clostridium sensu stricto 1, and Enterococcus genera in colon contents by diagnosis; P-value was determined using a Wilcoxon rank sum exact test; **:p-value ⩽ 0.01 healthy (n=15) vs NEC (n=31) (**a**). (**b**) Relative abundance of Bifidobacterium, Escherichia-Shigella, Clostridium sensu stricto 1, and Enterococcus genera in colon contents by treatment group; P-value was determined using Kruskal-Wallis multiple comparisons followed by p-value adjustment with the Benjamin-Hochberg method; **:p-value ⩽ 0.01 Form and Form3SL vs DHM (b). (DHM (n=9), Form (n=8), Form3SL (n=9), FormBI (n=11), and FormBoth (n=10)). (**c**) Spearman correlation of relative abundance of Bifidobacterium, Escherichia-Shigella, Clostridium sensu stricto 1, and Enterococcus with total NEC severity score (n=46).

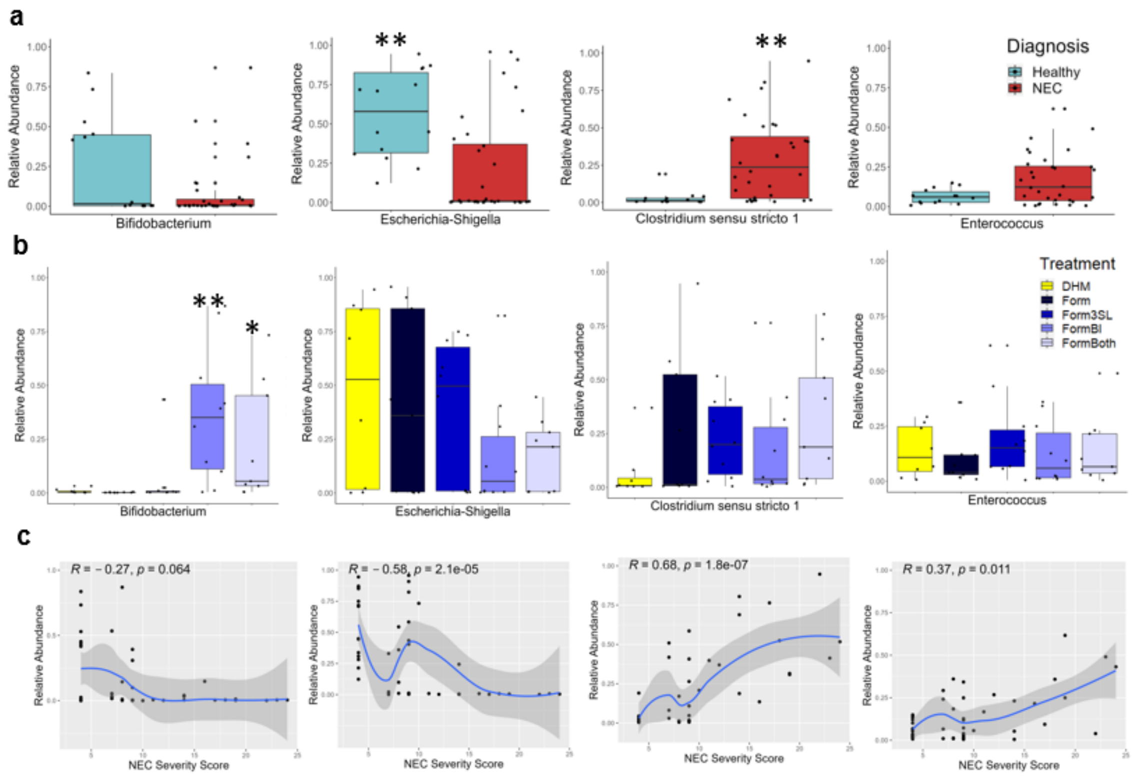

Supplementary Figure S7. Relative abundance and correlation with disease severity of top 3 most abundant genera in small intestinal contents by 16S sequencing. (**a**) Relative abundance of Bifidobacterium, Escherichia-Shigella, Clostridium sensu stricto 1, and Enterococcus genera in colon contents by diagnosis; P-value was determined using a Wilcoxon rank sum exact test; **:p-value ⩽ 0.01 healthy (n=15) vs NEC (n=31) (**a**). (**b**) Relative abundance of Bifidobacterium, Escherichia-Shigella, Clostridium sensu stricto 1, and Enterococcus genera in colon contents by treatment group; P-value was determined using Kruskal-Wallis multiple comparisons followed by p-value adjustment with the Benjamin-Hochberg method; **:p-value ⩽ 0.01 Form and Form3SL vs DHM (b). (DHM (n=9), Form (n=8), Form3SL (n=9), FormBI (n=11), and FormBoth (n=10)). (**c**) Spearman correlation of relative abundance of Bifidobacterium, Escherichia-Shigella, Clostridium sensu stricto 1, and Enterococcus with total NEC severity score (n=46).
